# Editorial Comment to False‐positive ^123^I‐metaiodobenzylguanidine scan in a patient with renal cell carcinoma: A case of chromophobe renal cell carcinoma oncocytic variant with a complicated clinical course

**DOI:** 10.1002/iju5.12244

**Published:** 2020-12-03

**Authors:** Naoto Kuroda

**Affiliations:** ^1^ Department of Diagnostic Pathology Kobe Kyodo Hospital Kobe Hyogo Japan

Suyama *et al*. reported a rare case of chromophobe renal cell carcinoma (RCC) oncocytic variant showing false‐positive for MIBG examination.^1^ Interestingly, there is no description on false‐positive MIBG scan in RCC.

Chromophobe RCC oncocytic variant is an extremely rare renal neoplasm and there are such previously reported six cases.^2^, ^3^ Additionally, Meigs syndrome associated with RCC seems to be a rare phenomenon. A case of non‐metastatic RCC with contralateral pleural effusion was previously described.^4^ This case had no ovarian tumor using computed tomography scan. Tessemer *et al*. reported a case of nonmetastatic urothelial carcinoma with pleural effusion and without ovarian tumor.^5^ However, a case reported by Suyama *et al*. seems to be the first RCC associated with true Meigs syndrome. In order to elucidate the mechanism showing false‐positive MIBG examination in RCC, further accumulation will be desirable.

## Conflict of interest

The author declares no conflict of interest.
